# Evaluation of chemical constituents and in vitro antimicrobial, antioxidant and cytotoxicity potential of rhizome of *Astilbe rivularis* (Bodho-okhati), an indigenous medicinal plant from Eastern Himalayan region of India

**DOI:** 10.1186/s12906-019-2621-6

**Published:** 2019-08-05

**Authors:** Vijeta Rai, Anoop Kumar, Vaskar Das, Shilpi Ghosh

**Affiliations:** Department of Biotechnology, University of North Bengal, Raja Rammohunpur, Darjeeling, West Bengal 734013 India

**Keywords:** *Astilbe rivularis*, Antibacterial, DPPH assay, Fluorescent probe, MTT assay, Cytotoxicity, Active compounds

## Abstract

**Background:**

*Astilbe rivularis* **L.** is an indigenous medicinal plant growing in high altitude of Darjeeling Himalayan region of India and Nepal. The plant rhizome has been used traditionally as medicine by local tribes to treat various ailments including infectious and other diseases. The present study aims to evaluate the plant rhizome for chemical composition and in vitro antioxidant, antibacterial and cytotoxic bioactivities.

**Methods:**

The methanolic extract of rhizome was analyzed for phytochemical constituents by biochemical and GC-MS methods. The antibacterial property of the extract was monitored by agar well diffusion assay. Antioxidant potential was assessed by in vitro DPPH and ABTS scavenging assays and scavenging of induced ROS in normal cell line using fluorescent probe 2′, 7′- dichlorofluorescin diacetate. Cytotoxic effect of the extract in cancer and normal cell lines was determined by MTT assay.

**Results:**

Rhizome methanolic extract contained terpenoids, flavonoids, tannins, phenols, alkaloids, saponins and reducing sugars. Further analysis of extract by GC-MS showed the presence of nine major constituents belonging to terpenoids and fatty acid groups. The extract had marked in vitro ROS scavenging activity and moderate antibacterial activity against gram positive and gram negative bacteria. It showed cytotoxicity to neuroblastoma (SHSY5Y) cell line with IC_50_ value < 100 μg ml^− 1^ but had least damaging effect on normal cells, like human embryonic kidney (HEK-293) and liver (WRL-68) cell lines.

**Conclusion:**

The study suggests that *Astilbe rivularis* has potential as source of new potent antibacterial, antioxidant and anticancer agents. Further studies on purification and characterization of active compounds from *Astilbe rivularis* and their biological evaluation are highly recommended.

## Background

Respiration is an important metabolic process of biological combustion to generate energy; however, the process also produces harmful intermediates called reactive oxygen species (ROS). The excessive accumulation of ROS leads to cumulative damage of biomolecules, such as proteins, lipids and nucleic acid, resulting in oxidative stress. Oxidative stress has been associated with several disease conditions such as diabetes, stroke, cancer, arteriosclerosis, alzheimer’s disease, cardiovascular diseases and ageing [1, 2].

Plants are being used as therapeutics from ancient time without the prior knowledge of their active components. They have been the major source of phytochemicals with therapeutic potential. These phytochemicals also act as reducing agents to reverse oxidation by donating electrons and/or hydrogen ions. Moreover, natural compounds as drugs have reduced side effects due to their regular intake as components of vegetable food. Scientific studies on ethnomedicinal plants have resulted in discovery of several therapeutic drugs [3, 4]. Previous research works on anticancer potential of plants resulted in the development of valuable anticancer drugs, such as taxol, camptothecin, vincristine and vinblastine [5]. These drugs are reported to target mitotic spindle assembly, chromosome segregation, cell division and apoptosis [6]. In recent years, natural anticancer compounds have been reported with various targeting mechanisms, like up-regulation of p16INK4A, preventing MRCK- kinase that targeting multiple gene products and targeting mitotic processes in different types of cancer, such as human mouth epidermal carcinoma, murine leukemia, human colorectal cancer and prostate cancer [7].

*Astilbe*, a genus with 18 species of rhizomatous flowering plants of the family Saxifragaceae, is native to mountain ravines and woodland in Asia and North America [8]. It is a traditional medicinal plant used by ethnic people of Eastern Himalayan region of India and Nepal. Almost all parts of the plant are being used as medicine with most preferential use of the rhizome part. Although the plant has shown antiviral, antidiabetic and antiulcer properties, little scientific information are available on its efficacy as drug source. The plant extract has shown antiviral effect against Herpes Simplex Virus [9]. Pentacyclic triterpenoids isolated from the *Astilbe* plant enhanced glucose uptake via the activation of Akt and Erk1/2 in C2C12 myotubes [10]. Moreover, a compound (AR-I) isolated from the plant leaves showed antiulcer activity against ethanol induced gastric ulcer and cysteamine induced duodenal ulcer in albino rats [11].

Our survey on the use of medicinal plants by tribes and local inhabitants of hilly area of Darjeeling revealed the therapeutic importance of rhizome of *Astilbe rivularis* (AR) in reducing various types of infections as well as diabetes. Therefore, in the present study, the methanolic extract of the rhizome was investigated for phytochemical classes followed by identification of major phytoconstituents by GC-MS. AR rhizome extract showed in vitro antioxidant and antibacterial activities. The extract had considerably greater cytotoxic effect against Neuroblastoma cell line (SHSY5Y) compared to normal cells, like Human Embryonic Kidney (HEK-293) and Liver (WRL-68) cell lines. To our knowledge, this is the first study of its kind on AR where the plant showed multiple bioactivities and thus can be a potential candidate for pharmaceutical industries.

## Materials and methods

### Plant material, human cell lines and chemicals

*Astilbe rivularis* rhizome was collected from Jalapahar Cantonment Forest situated at high altitude of Darjeeling Himalayan region (27^o^ 2′9.6252 N and 88^o^ 15′ 45.6192 E) of West Bengal, India, during the month of February, 2016. The plant was authenticated by Dr. Monoranjan Chowdhury, Assistant Professor, Department of Botany, University of North Bengal, Siliguri, India. A voucher specimen (NBU-Bot/10045/2016) has been deposited in the institutional herbarium for future reference. Neuroblastoma (SHSY5Y), Human Embryonic Kidney (HEK-293) and Liver (WRL-68) cell lines were obtained from National Centre for Cell Science, Pune, India. All other chemicals of analytical grade were purchased from Sigma-Aldrich India Limited, Hi Media, India and E. Merck, India.

### Preparation of plant extract

The plant rhizome was dried in shade and then ground into fine powder. Methanolic extract of the rhizome was prepared by Soxhlet extraction method. About 20 g of powdered plant material was uniformly packed into a thimble and extracted with 200 ml of methanol for 24 h or till the solvent in siphon tube of extractor became colorless. The extract obtained was concentrated under reduced pressure in a rotary evaporator to fine powder. The total yield percentage of the plant extract was 39.75% as calculated by the formula, Yield Percentage (%) = (Weight of extract obtained)/ (Total weight of sample loaded) × 100.

### Qualitative analysis of phytochemicals

The methanolic rhizome extract was screened for phytochemicals, such as phenols, flavonoids, tannins, saponins, terpenoids, cardiac glycosides and alkaloids. For phenol detection, 100 mg of powdered plant sample was suspended in 5 ml double distilled water, mixed well and filtered through Whatman No.1 filter paper. To 1 ml of filtrate equal volume of 1% FeCl_3_ was added. Appearance of blue or green color confirmed the presence of phenol [12]. For detection of flavanoid**,** 1 g sample was mixed with 5 ml of acetone, followed by evaporation of acetone in hot water bath. The precipitate was extracted with 5 ml of warm double distilled water, filtered under hot condition and cooled at room temperature (RT). To 1 ml of the filtrate equal volume of 20% NaOH was added. Appearance of yellow colour indicated the presence of flavonoids [13]. The methanolic filtrate of plant rhizome powder (100 mg ml^− 1^) was used for detection of alkaloid, terpenoid, cardiac glycoside and saponin. Methanolic filtrate (2 ml) was mixed with 2 ml of 1% HCl and kept over steam for 5 min. To 1 ml of mixture, 6–7 drops of Mayer’s/Wagner’s reagent was added to obtain creamish/ brown/ red/ orange precipitate indicated the presence of alkaloids [14]. 1 ml of methanolic filtrate was mixed with 1 ml of chloroform, 1 ml of acetic anhydride and 0.5 ml of concentrated H_2_SO_4_. Appearance of reddish brown coloration at the interface indicated the presence of terpenoid [15]. 1 ml methanolic filtrate was mixed with 0.5 ml glacial acetic acid followed by addition of 3–4 drops of 5% FeCl_3_ and 0.5 ml of concentrated H_2_SO_4_. Appearance of brown ring at the interface indicated presence of cardiac glycosides [14]. For detection of tannins and saponins, 100 mg rhizome powder was suspended in 5 ml of double distilled water and filtered. To 0.5 ml of aqueous filtrate, 5 ml double distilled water was added and shaken vigorously for about 30 s. Formation and persistence of froth indicated the presence of saponins [14]. To 2 ml filtrate, 1 ml of 5% FeCl_3_ was added. The formation of yellow brown precipitate confirmed the presence of tannins [16].

### GC-MS analysis

The chemical constituents of methanolic extract of *Astilbe* rhizome were identified by GC-MS analysis on GC-MS JEOL, GC Mate II equipped with HP5 silica column (50 m × 0.25 mm i.d.) and secondary electron multiplier. The sample (1 μl) was evaporated in a splitless injector at 300 °C. The analysis conditions were 20 min at 100 °C, 3 min at 235 °C for column temperature and 240 °C for injector temperature. Helium was the carrier gas and split ratio was 5:4. Run was carried out for 22 min. The chemical constituents of the rhizome extract were identified by the comparison of the experimental mass spectra with that of National Institute Standard and Technique (NIST) GC-MS database [17].

### DPPH free radical scavenging assay

Antioxidant property of the rhizome extract was determined by its ability to reduce purple-colored methanolic solution of DPPH (2, 2-diphenyl-1-picryllhydrazyl) free-radical to colourless compound [18]. The extract at various concentrations (5–30 μg ml^− 1^) was mixed with equal volume of methanolic DPPH solution (100 μM), incubated at RT for 30 min in dark and absorbance was recorded at 517 nm. The reaction mixture without extract served as control and L-ascorbic acid was used as antioxidant standard. The percentage inhibition was calculated according to the formula: DPPH inhibition (%) = (A_o_ – A_1_)/A_o_ × 100, where A_o_ and A_1_ are absorbance of control and extract/standard, respectively.

### ABTS scavenging assay

ABTS scavenging assay was performed by standard method with some modifications [19]. Briefly, ABTS stock solution was prepared by mixing potassium per sulfate (2.45 mM) and ABTS (7 mM) in equal ratio and incubating at RT in dark for 12–16 h. The working ABTS^+^ solution was prepared by dilution of the stock solution with 80% methanol to absorbance A_734_ = 0.708 ± 0.002. Plant extract (10 μl) at concentrations ranging from 25 to 200 μg ml^− 1^ in methanol was added to 1 ml of ABTS^+^ working solution. Thereafter, the reaction mixture was incubated at RT for 7 min in dark and absorbance was recorded at 734 nm. The reaction mixture without extract served as control, whereas L-ascorbic acid and gallic acid served as antioxidant standards. The percentage inhibition was calculated using the formula: ABTS scavenging (%) = (A_o_ – A_1_)/A_o_ × 100, where A_o_ and A_1_ absorbance of control and extract/standard, respectively.

### Evaluation of rhizome extract for scavenging of induced ROS in WRL-68 cells using fluorescent probe 2′, 7′- dichlorofluorescin diacetate (DCF-DA)

The coverslips seeded with cells (WRL-68) at concentration of 2.5 × 10^4^, were placed over 35 mm petriplates and grown overnight in the Dulbecco’s Modified Eagle Medium (DMEM), supplemented with 5% fetal bovine serum (FBS), 100 IU ml^− 1^ Penicillin and 100 μg ml^− 1^ of Streptomycin at 37 °C in 5% CO_2_ incubator. The culture plates were divided into four sets and three sets were treated with incomplete medium (DMEM without FBS) and one set was treated with complete medium (DMEM with FBS). Out of three sets of cells grown in incomplete media, two sets were treated with 25 μl of methanolic extract at 50 and 100 μg ml^− 1^ concentrations, whereas third set treated with 25 μl of methanol served as control. The untreated cells grown in complete media served as the positive control. The intracellular oxidative level was examined using the dichlorofluorescein assay [20, 21]. For this both treated and untreated cells were washed with phosphate-buffer saline (PBS) followed by addition of 10 μM carboxy-2′, 7′–dichlorofluorescin diacetate (DCF-DA) and incubated for 30 min at 37 °C in dark in an incubator (5% CO_2_). Cells washed with PBS were immediately analyzed for generation of ROS under fluorescence microscope (Magnus MLXi, Olympus).

### Determination of antibacterial activity

The antibacterial property of the rhizome extract was monitored by agar well diffusion method using Mueller Hinton Agar (MHA) medium [22]. Bacterial culture grown overnight in Muller Hinton broth at 37 °C was swabbed into the surface of MHA plates. Wells were prepared on the plates with the help of sterile 6 mm cork-borer and the (30 μl) plant extract of different concentrations (20–100 mg ml^− 1^) introduced into the wells followed by incubation of plates at 37 °C for 24 h and thereafter, the zone of inhibition was measured. Antibiotics ampicillin (2 μg ml^− 1^) and tetracycline (30 μg ml^− 1^) were used as standards and extraction solvent (methanol) was used as control.

### Cytotoxicity analysis

Cytotoxic activity of the plant rhizome extract against normal and cancer cell lines was determined by using standard MTT [(3-(4, 5-dimethylthiazol-2-yl)-2,5-diphenyltetrazolium bromide] assay [23]. For this purpose, SH-SY5Y, HEK-293 and WRL-68 were separately cultured in DMEM, supplemented with 5% FBS, 100 IU ml^− 1^ Penicillin and 100 μg ml^− 1^ of Streptomycin, in 100 mm petri dishes at 37 °C in 5% CO_2_ incubator. 100 μl of cells (1 × 10^5^ cells ml^− 1^) were seeded into each well of 96 well microplate. After 24 h incubation, cells were treated with specified concentrations of plant extract. After 24 h of treatment, 10 μl of MTT (5 mg ml^− 1^) was added to each well and incubated further for 4 h. The MTT was replaced by 50 μl isopropanol to dissolve the insoluble formazan product. The extent of MTT reduction to formazan within the cells was calculated by measuring the absorbance at 540 nm using a micro plate reader (Spectrostar^nano^, BMG Labtech). The inhibition of cell growth was calculated by the formula; Percent inhibition (%) = (Y-X)/Y × 100, where Y is the mean optical density of control (DMSO treated cells) and X is the mean optical density of cells treated with plant extract. For cytotoxicity assay, the stock solution of extract was prepared in DMSO, which was diluted to final desired concentrations using the same solvent.

### Statistical analysis

All experimental results are mean ± SD of three parallel measurements. The data were analyzed by analysis of variance (*P* < 0.05). Results were processed in Excel and SPSS programmes.

## Results

**Fig. 1:**
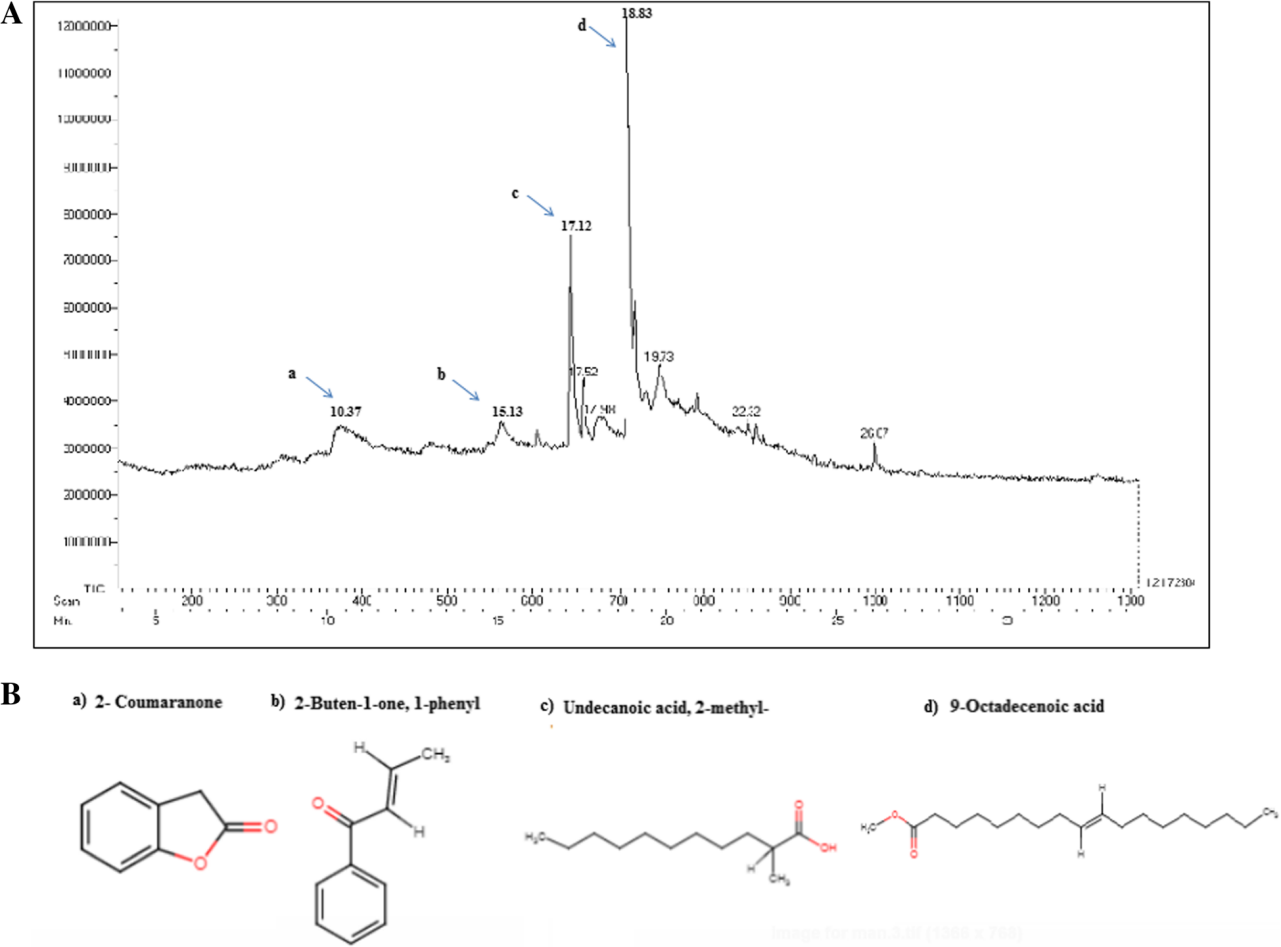
GC-MS analyses of AR rhizome. (**a**) GC/MS chromatogram of *Astibe rivularis* rhizome analyzed on GC system. It showed the presence of nine compounds. (**b**) Chemical structures of the active compounds are shown here

The preliminary in vitro phytochemical analysis of methanolic extract of AR rhizome revealed the presence of various classes of phytochemicals, such as tannins, phenols, cardiac-glycosides, saponins, terpenoids and alkaloids with terpenoids being the most abundant one (Table 1). Further the qualitative and quantitative analyses of phytochemicals were performed by GC-MS. The chromatogram showed nine prominent peaks based on their retention time indicating the presence of nine major phytochemical constituents (Fig. 1). The structure, molecular formula, molecular weight and percent peak area of these compounds are mentioned in Table 2.

**Table 1:** In vitro phytochemical analysis of the AR rhizome extract

Phytochemical	Inference/Result
Tanins	+
Phenols	+
Flavanoids	+
Terpenoids	++
Alkaloids	+
Saponins	+
Cardiac glycosides	–
Reducing sugars	+

‘+‘denotes presence of phytochemical

‘-‘denotes absence of phytochemical

**Table 2:** List of major phytoconstituents identified in the methanolic extract of AR (rhizome) by GC-MS

*S.No*	RT	Name of the compound	Molecular Formula	MW	Peak Area %
1	10.37	2-Coumaranone	C_8_H_6_O_2_	134.13	28.4
2	15.13	2-Buten-1-one, 1-phenyl	C_11_H_12_O	146.18	20
3	17.12	Undecanoic acid, 2-methyl	C_12_H_24_O_2_	200.31	10.6
4	17.52	2-Piperidinone, 3,6-bis (1-methyllethenyl)-1-phenyl,trans	C_17_H_21_NO	255.36	4.4
5	17.98	Crinan 1,2-didehydro	C_17_H_19_NO_4_	301.34	1.7
6	18.83	9-Octadecenoic acid (z)-,methyl ester	C_19_H_36_O_2_	296.49	29.8
7	19.73	[1,1-Bicyclopropyl]-2-octanoic acid, 2-hexyl-, methyl ester	C_21_H_38_O_2_	322.53	3.6
*8*	22.32	17a-Ethyl-3a-methoxy-17a-aza-D-homoandrost-5-ene-17- one	C_21_H_33_NO_2_	331	1.1
*9*	26.07	Butanedioicacid, 2,3-bis(8-nonen-1-yl)-, dimethyl ester	C_24_H_42_O_4_	394.58	0.42

**Fig. 2:**
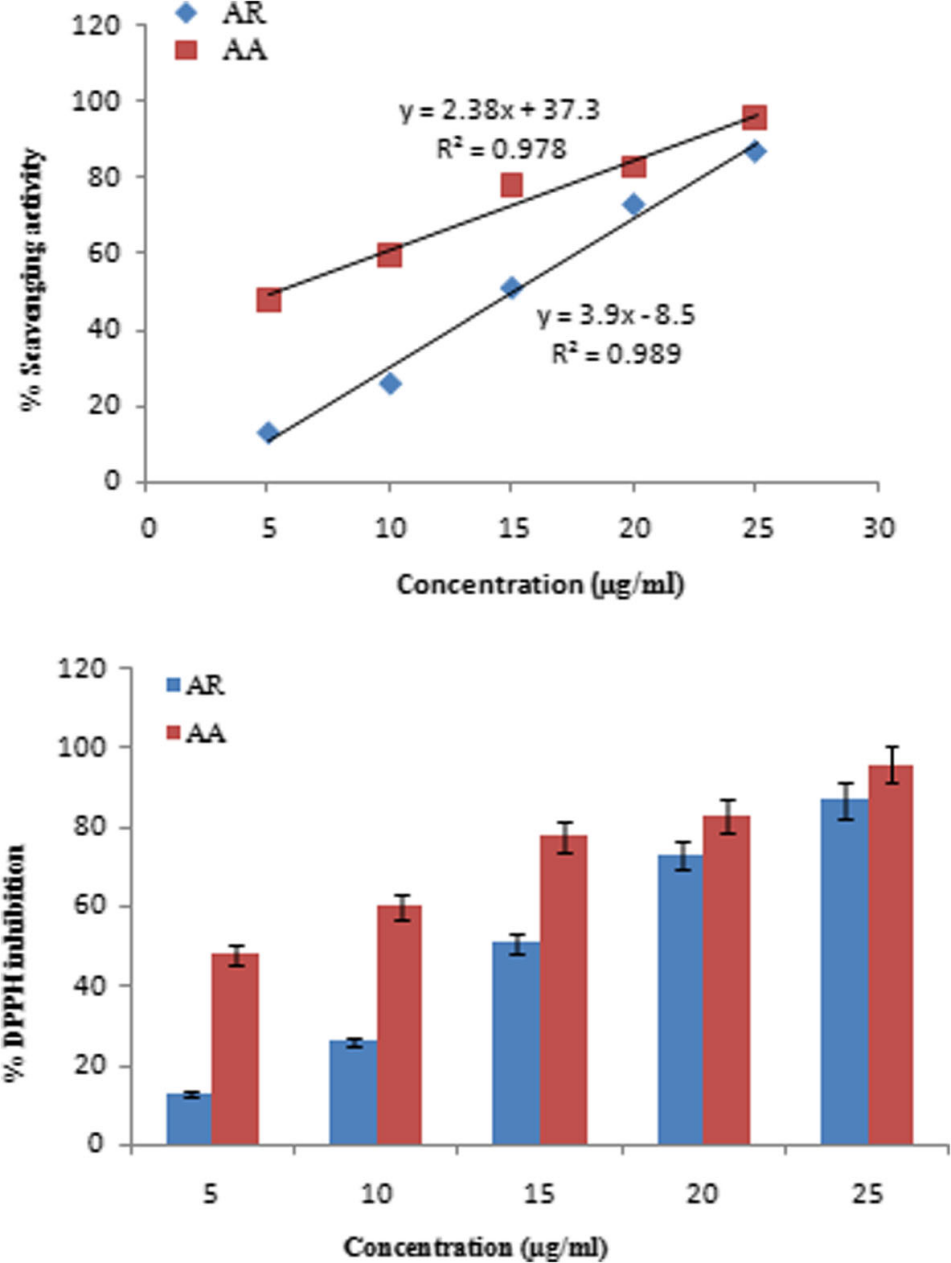
Scavenging of DPPH free radical by AR rhizome extract. **a**) The linear regression curve and **b**) Percent of DPPH inhibition. Data represent the mean of three replicates. Ascorbic acid was used as standard. **AR- *Astilbe rivularis,* AA- Ascorbic acid

**Fig. 3:**
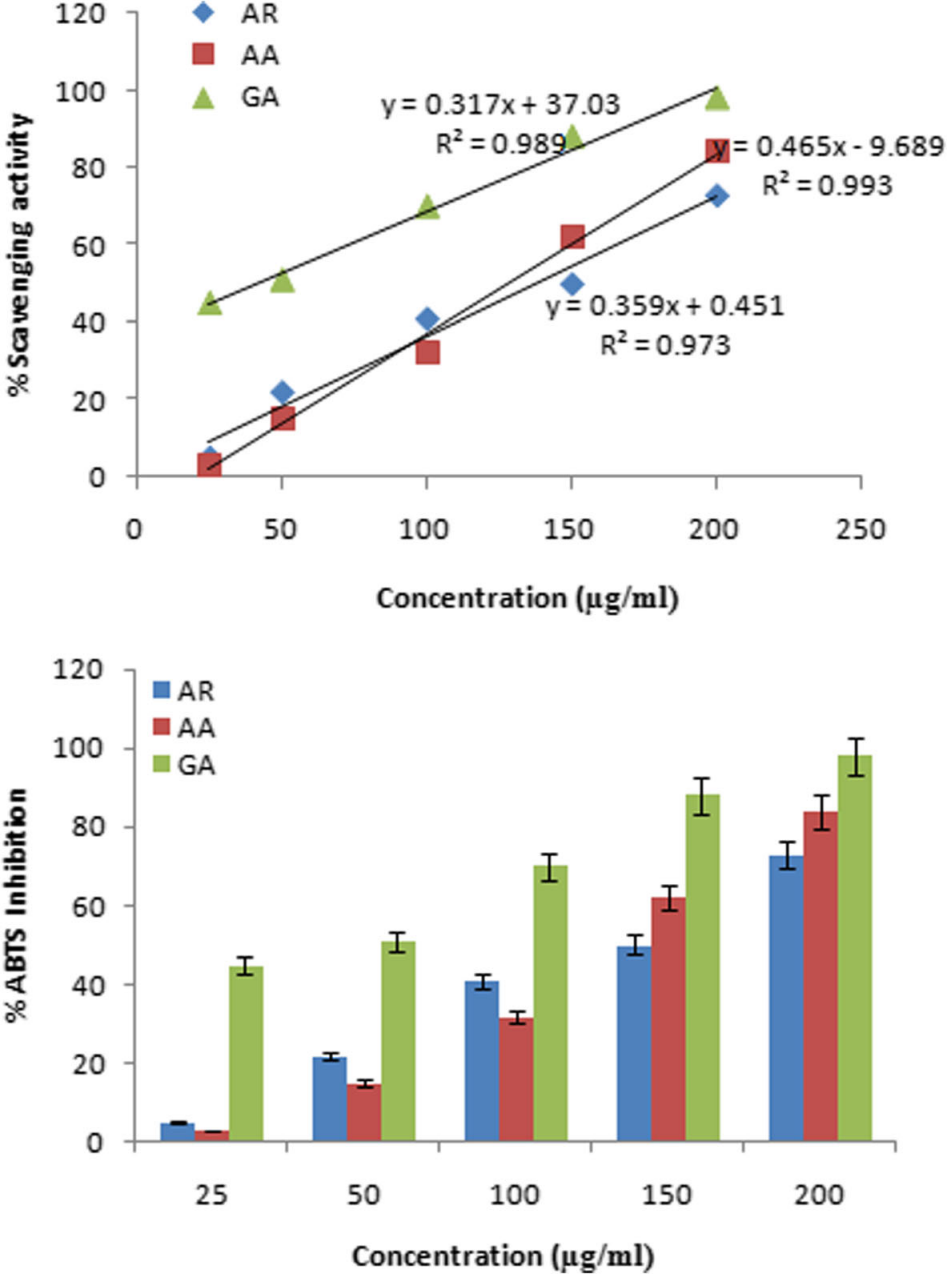
Scavenging of ABTS free radical by AR rhizome extract **a**) The linear regression curve and **b**) Percent of ABTS inhibition. Data represents the mean of three replicates. Ascorbic acid and Gallic acid were used as standard. **AR- *Astilbe rivularis,* AA- Ascorbic acid, GA- Gallic acid

**Fig. 4:**
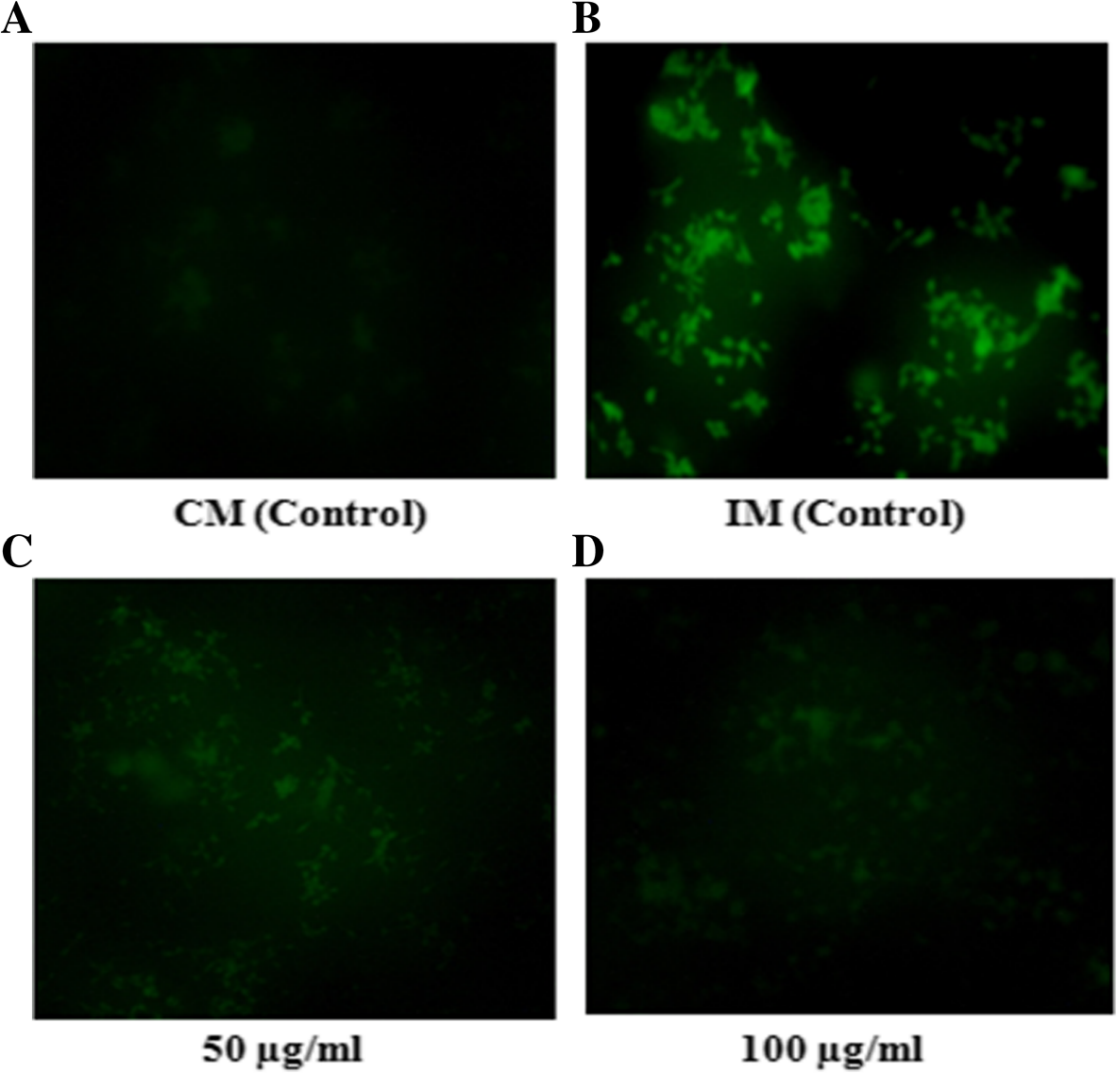
Effects of AR rhizome extract on oxidative stress in the WRL-68 cells. **a**) Unstressed cells grown in CM showed basal level ROS **b**) Control cells grown in IM showing increased ROS generation **c**) Cells in IM treated with 50 μgml^− 1^ of extract **d**) Cells in IM treated with 100 μgml^− 1^ of extract. **CM- Complete Media (DMEM with FBS), IM- Incomplete Media (DMEM without FBS)

The methanolic extract was analyzed for antioxidant activities based on different working principles. AR rhizome showed the ability to scavenge DPPH free radical in a concentration dependent manner; however, its scavenging capacity was lower than that of the standard antioxidant ascorbic acid (Fig. 2). Consequently, the IC_50_ values as calculated from linear regression analysis curve were 15 and 5 μg ml^− 1^ for rhizome extract and ascorbic acid, respectively. ABTS free radical scavenging potential of AR and of the standard antioxidants such as ascorbic acid and gallic acid, were observed at different concentrations i.e. 25, 50, 100, 150 and 200 μg ml^− 1^ and their IC_50_ values were 138, 128 and 40 μg ml^− 1^, respectively (Fig. 3). 6-carboxy-2′, 7 dichlorodihydrofluorescein diacetate (carboxy-H2DCFDA) is a non-fluorescent reagent, which in presence of free radicals undergoes oxidation to produce green fluorescence. Although it mainly detects peroxides, it also gets oxidized by other ROS generated in the cells due to stress [24]. In our study, the normal liver cell line (WRL-68) grown in incomplete media led to production of ROS, that reacted with carboxy-H2DCFDA to give intense fluorescent signal (Fig. 4b). However, the level of ROS generation on growing in incomplete medium significantly reduced in the presence of various doses of plant extract. The results in Fig. 4c and d demonstrate the dose dependent lowering of fluorescent signal in the cells on exposure to the crude extract at 50 and 100 μg ml^− 1^ as compared to untreated control (Fig. 4a).

Antibacterial effect of AR extract was evaluated against five different bacteria, including *Bacillus subtilis*, *Bacillus amyloliquefaciens*, *Aeromonas liquefaciens*, *Flexibactor sp.* and *Psedomonas sp.*, by agar well diffusion method and zone of inhibition (ZOI) formed on the plates was measured. From the results in Table 3 it is evident that the rhizome extract showed antibacterial effect against both gram +ve and gram –ve bacteria. The extract at 20–100 mg ml^− 1^ concentration produced ZOI in the range of 13–24 mm (*P* < 0.001) with the highest ZOI of 23 and 24 mm against *A. liquefaciens* and *B.amyloliquefaciens* at 100 and 80 mg ml^− 1^, respectively.

**Table 3:** Antimicrobial effect of AR rhizome extract at different concentrations

Extract concentration and Zone of inhibition (mm) Standard antibiotics								
Plant	Microbes	20 mg/ml	40 mg/ml	60 mg/ml	80 mg/ml	100 mg/ml	^a^Amp.	^b^Tet.
*Astilbe rivularis*	*B.subtilis*	15	16	16	17	17	25	35
	*B.amyloliquefaciens*	16	20	23	24	24	27	35
	*A.liquefaciens*	13	18	18	20	23	30	40
	*Flexibactor sp*.	14	17	18	20	20	18	34
	*Pseudomonas sp.*	-	–	12	14	17	24	33

^a^Amp. - Ampicillin at 2 μg ml^−1^ concentration

^b^Tet. – Tetracyclin at 30 μg ml^−1^ concentration

**Fig. 5:**
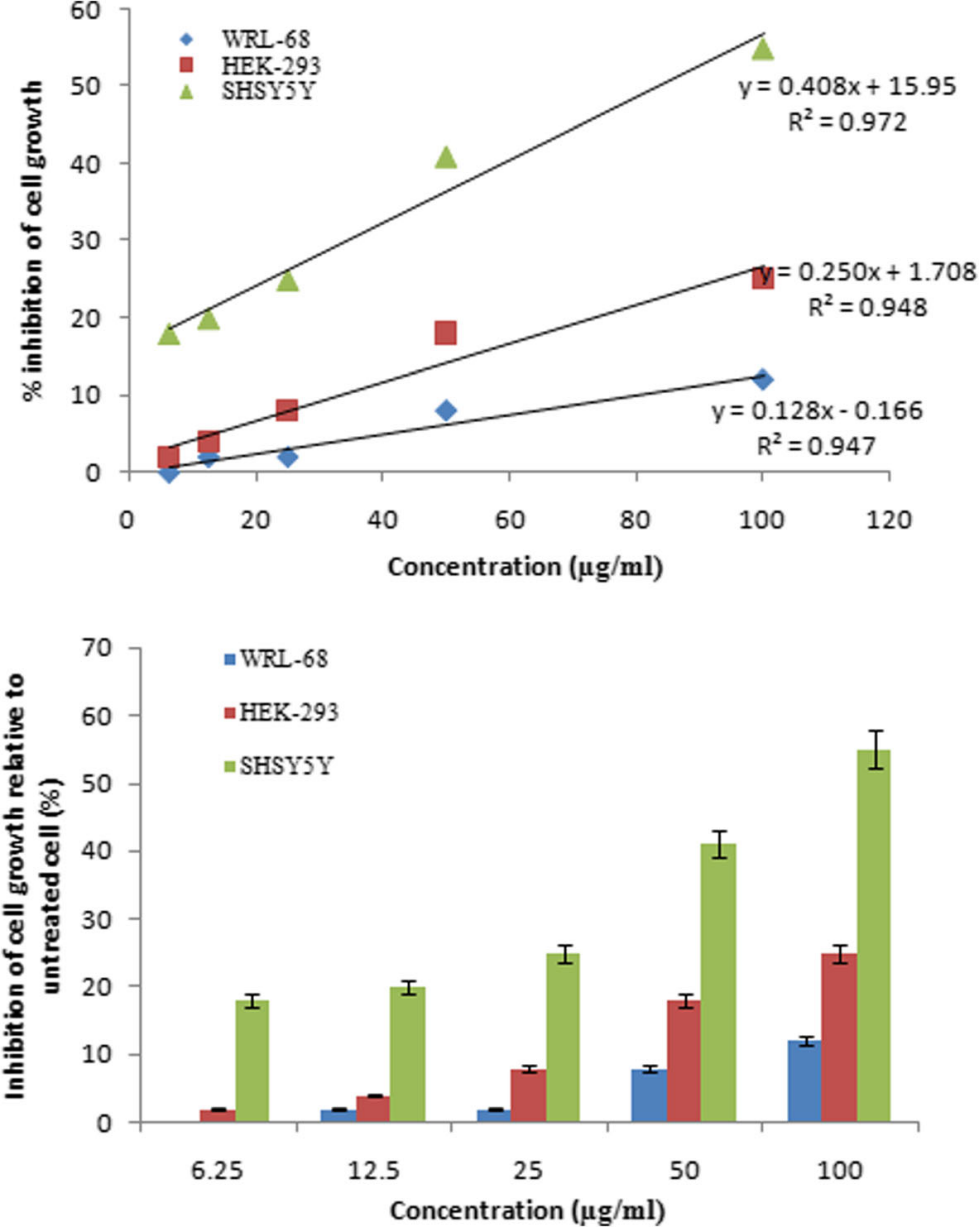
Cytotoxicity effects of AR rhizome extract  against the Neuroblastema cell (SHSY5Y), Human Embryonic kidney cell (HEK- 293) and Liver cell line (WRL-68). Data represent the mean of three replicates

The plant extract also showed cytotoxic effect against neuroblastema cell line (SHSY5Y) and two normal cell lines namely, HEK-296 and WRL-68, with IC_50_ value of 83.7, 193.8 and 389.3 μg ml^− 1^, respectively (Fig. 5). The results thus indicate a relatively greater inhibition of cancer cells proliferation by the active components of the plant rhizome extract.

## Discussion

The rhizome of *A. rivularis* is ethanopharmacologically known to treat a number of ailments including stomach ache, diarrhoea, dysentery, headache, cough, rheumatism, back pain, wound healing, weakness, avian plague, peptic ulcer and malaria [25, 26]. Moreover, powdered root is taken with curd to cure jaundice and with honey to control excessive bleeding after child birth [27]. However, the plant has not been explored much for identification of phytoconstituents and their biological activities. Hence, in the present work methanolic extract of the rhizome was analyzed for phytoconstituents, antimicrobial and antioxidant activities, and in vitro cytotoxicity against normal and cancer cell lines. Primarily, plant water extracts are being used for their medicinal use, however, plants extracted in organic solvents have been found to give more consistent in vitro biological activities [22, 28]. Therefore, in our study we used methanol as polar organic solvent for extraction of *A. rivularis* rhizome. Phytochemical assay of the rhizome extract showed the presence of terpenoids, alkaloids, tannins, flavanoids and phenols that are known for several bioactive functions, such as antioxidant, antimicrobial and anticancer activities. The qualitative analysis by GC-MS identified different types of compounds that include 2-coumaranone; 2-buten-1-one, 1-phenyl; Undecanoic acid, 2-methyl; 2-piperidinone, 3,6-bis (1-methyllethenyl)-1-phenyl,trans; Crinan 1,2-didehydro; 9-Octadecenoic acid (z)-,methyl ester; [1,1-bicyclopropyl]-2-octanoic acid, 2-hexyl-, methyl ester; 17a-ethyl-3a-methoxy-17a-aza-D-homoandrost-5-ene-17- one and Butanedioic acid, 2,3-bis (8-nonen-1-yl)-, dimethyl ester. In a previous study, coumaranone terpenoid isolated from *Astilbe* species has been found to inhibit acetyl choline esterase [29]. The compound also showed antidiabetic effect by enhancing glucose uptake via activation of Akt and Erk1/2 in C2C12 myotubes. Undecanoic methyl esters are reported to have cytotoxic effect against breast, ovarian, prostrate and liver cancer cell lines with IC_50_ values in the range of 10–140 μM [30].

The incidence of drug resistance against microorganisms is a leading cause of ineffectiveness of antimicrobial agents. Medicinal plants could act as potential source of new antibacterial agents even against some resistant strains of microorganisms. In earlier research works, several plant extracts were found to be more effective against gram positive bacteria than gram negative ones because of the presence of impermeable lipopolysacharide layer [31]. In the present study, methanolic AR rhizome extract was found to be effective against both gram +ve and gram -ve bacteria and with greater ZOI at higher extract concentrations. In an earlier study, the compounds arbutin and bergenin isolated from methanolic extract of rhizome of *A. rivularis* inhibited the growth of *E.coli* [25, 32].

Phytochemicals are considered to have multiple beneficial effects through neutralization of free radicals associated with several diseases [33]. They neutralize the free radical by either donating hydrogen or quenching singlet oxygen. *Astilbe* rhizome extract showed antioxidant activity, with a more effective scavenging of DPPH as compared to that of ABTS. In an earlier research on *A. rivularis*, the methanolic leaf extract exhibited 96% DPPH scavenging activity at a dose of 100 μg ml^− 1^ [34], whereas we found almost similar level of DPPH scavenging at 25 μg ml^− 1^ of methanolic rhizome extract. The results thus indicate markedly greater antioxidant potential of rhizome compared with leaf. Even though the scavenging activities of the extract were significantly lower in comparison to standards (gallic acid and ascorbic acid), the reduced activities could be related to the lesser amounts of antioxidant compounds in the extract [22]. GC-MS analysis of *Astilbe* rhizome extract indicated the presence of fatty acid ester i.e. methyl ester of 9-Octadecenoic acid as one of the most abundant molecule with 29.8% peak area, which has also been earlier reported of having reducing potential [3].

Serum deprivation in the growth medium has been reported to trigger cellular ROS generation [35]. Hence in our study, ROS production was induced by growing the normal liver cell line in serum deprived medium, which was effectively lowered down in presence of the plant extract.

The in vitro cytotoxic activity of plant extracts with IC_50_ < 100 μg ml^− 1^ is generally considered to be therapeutically important [36]. The rhizome extract exhibited in vitro cytotoxic activity for cancer cell line i.e. SHSY5Y, with IC_50_ < 100 μg ml^− 1^, whereas IC_50_ > 100 μg ml^− 1^ was obtained for normal cell lines. The results thus suggest *A. rivularis* rhizome extract as a potential candidate for cancer research.

## Conclusion

The methanolic extract of *A. rivularis* rhizome showed the presence of various phytochemical constituents, such as terpenoids, alkaloids, tanins and phenols. Further the GC-MS analysis of the extract confirmed the presence of some antibacterial and anticancer compounds. The extract was the source of effective antioxidants as revealed by DPPH, ABTS and DCF-DA based ROS scavenging assays and of effective antimicrobial compounds against both gram positive and gram negative bacteria. *Astilbe* rhizome extract also exhibited moderate cytotoxicity against the cancer cell line with limited effect on the normal cell lines. The results suggest that *A. rivularis* has the potential as therapeutic agent for disease conditions. Further, the study also validates its traditional use by ethnic people. Hence, our further research work has been directed towards the isolation of pure compound(s) from the methanolic rhizome extract of *A. rivularis* and evaluation of their biological activity and mechanism of action.

## Data Availability

The datasets supporting the conclusions of this article are included within the article. The. Datasets/materials used and/or analyzed during the current study are available from the. corresponding author on reasonable request.
